# Antimicrobial resistance of bacteria isolated from blood cultures at a tertiary hospital in Papua New Guinea: implications for antimicrobial stewardship and surveillance

**DOI:** 10.3389/fpubh.2026.1914935

**Published:** 2026-10-12

**Authors:** Watson Toroi, Victor Musyoki, Becky Max, Donella Omena, Nomvuyo Mothobi, Kudakwashe Chani, Josephine Chanoan, Amrita Ronnachit

**Affiliations:** 1 Department of Pathology, Goroka Provincial Hospital, Goroka, Papua New Guinea; 2 AMR-One Health Working Group, Burnet Institute, Melbourne, VIC, Australia; 3 Department of Medical Microbiology and Immunology, University of Nairobi, Nairobi, Kenya; 4 Department of Infectious Diseases, Barwon Health, Geelong, VIC, Australia; 5 Microbiology Unit, Alfred Pathology Service, Footscray Hospital, Melbourne, VIC, Australia; 6 Australian HPV Reference Laboratory, Australian Centre for the Prevention of Cervical Cancer, Melbourne, VIC, Australia; 7 Royal Prince Alfred Hospital, Sydney, NSW, Australia; 8 Sydney Medical School, Faculty of Medicine and Health, Sydney University, Sydney, NSW, Australia

**Keywords:** antimicrobial resistance, antimicrobial stewardship, bacterial isolates, bloodstream infections, Fleming fund, Goroka, Papua New Guinea

## Abstract

**Introduction:**

Bloodstream infection (BSI) is a global healthcare concern, especially in low- and middle-income countries (LMIC) where diagnostic capacity and antimicrobial stewardship programs remain limited. Local data on bacterial causative agents and antimicrobial resistance (AMR) remain scarce in the Pacific Island Countries and Territories, including Papua New Guinea (PNG). This study aimed to determine the prevalence, bacterial spectrum, antimicrobial susceptibility patterns, and distribution of bloodstream pathogens among patients attending Goroka Provincial Hospital in Papua New Guinea.

**Methods:**

We conducted a retrospective laboratory surveillance study using routine blood culture data from Goroka Provincial Hospital. Bacterial identification and antimicrobial susceptibility results were retrieved from Senaite Laboratory Information Management System (LIMS) and analysed using WHONET and SPSS.

**Results:**

A total of 1,276 blood culture specimens were collected from patients with clinically suspected BSI, of which 141 (11.1%) yielded bacterial pathogens. *Salmonella enterica* servovar Typhi (*S.* Typhi) was the predominant isolate (72/141, 51.1%), followed by *Staphylococcus aureus* (*S. aureus*) (23/141, 16.3%). Most *S.* Typhi isolates were susceptible to first line antibiotics tested (98–100%), however, 45% were resistant to chloramphenicol. Methicillin-resistant *S. aureus* (MRSA) accounted for 60.9% of *S. aureus* isolates. Extended-spectrumβ-lactamase (ESBL)-positive *Escherichia coli* and *Klebsiella pneumoniae* were susceptible to meropenem, piperacillin-tazobactam, and amikacin.

**Conclusion:**

*Salmonella* Typhi was the predominant cause of BSI and showed substantial chloramphenicol resistance, while phenotypically ESBL-positive bacteria were also detected. These findings underscore the need for continued laboratory-based AMR surveillance and locally informed antimicrobial stewardship and infection-prevention strategies in PNG.

## 1 Introduction

Bloodstream infection (BSI) is a significant cause of morbidity and mortality globally (1). It occurs when microorganisms such as bacteria, fungi, or viruses from the environment or as part of the human microbiome enter the bloodstream to cause systemic infection. If not treated using appropriate antimicrobial agents and in a timely manner, BSI rapidly progresses to sepsis or life-threatening septic shock, with infants and the geriatric populations at a higher risk of severe infection (1, 2). Others at greater risk of severe and fatal infection include those who have recently been hospitalized, as well as immunosuppressed individuals, such as cancer patients (2). Globally, approximately 50 million people present with sepsis every year, with an estimated 11 million deaths reported annually. Of these, 2.9 million deaths occur among children under the age of five (3). According to the Global Burden of Disease study, BSI is among the leading underlying causes of sepsis, with high case numbers and deaths reported in LMICs (4). Several bacterial pathogens, including *Staphylococcus aureus (S. aureus)*, *Escherichia coli (E. coli)*, *Acinetobacter baumannii (A. baumannii)*, *Klebsiella pneumoniae (K. pneumoniae),* and *Streptococcus pneumoniae (S. pneumoniae),* have been isolated in sepsis cases and attributed to sepsis-related deaths (5). This underscores the importance of accurate identification of the pathogen to guide therapy for optimal clinical outcomes. However, the management of infectious diseases, including BSI, remains increasingly challenging due to the rising burden of antimicrobial resistance (AMR) (4). This silent pandemic has been associated with prolonged hospital stays, increased risk of hospital-acquired infection, and higher healthcare costs, especially in LMICs, where resources remain limited (6). According to the World Health Organization (WHO), third-generation cephalosporin-resistant and carbapenem-resistant Enterobacterales (3GCRE), methicillin-resistant *Staphylococcus aureus* (MRSA), and multidrug-resistant *Acinetobacter baumannii* are bacterial pathogens of major concern in healthcare (7). These organisms have been associated with both community- and hospital-acquired BSI and mortality (7, 8).

The burden of AMR and BSI associated with bacterial pathogens is poorly understood in a majority of LMICs. In Pacific Island countries, including Papua New Guinea (PNG), lack of local data to assess AMR and associated burden may be largely attributed to limited technical expertise and diagnostic capacity, lack of routine laboratory-based surveillance, and unreliable data management and reporting systems (2, 4). Moreover, high prevalence of infectious diseases in the region can be linked to a lack of standardized prescribing practices and reliance on outdated treatment guidelines that do not reflect local AMR data and trends (9). This contributes to selection of AMR strains and suboptimal clinical outcomes, including mortality, especially among neonates, geriatrics, and the immunosuppressed (6, 9–11).

To address these gaps, the UK aid-funded Fleming Fund Country Grant project, implemented by the Burnet Institute, has been supporting PNG to strengthen AMR surveillance and diagnostic capacity. The country has significant opportunities to further strengthen the generation and use of AMR data, including data from BSI, to inform evidence-based policy development, clinical decision making, and national strategies to combat AMR. The aim of this study was to determine the prevalence, bacterial spectrum, antimicrobial susceptibility patterns, and distribution of bloodstream pathogens among patients attending Goroka Provincial Hospital in Papua New Guinea.

## 2 Methods

### 2.1 Study design and setting

This was a retrospective observational laboratory-based surveillance study conducted using electronic laboratory records of bacterial isolates recovered from blood culture specimens collected from patients with clinically suspected BSI attending Goroka Provincial Hospital in Papua New Guinea. The study included blood culture data collected continuously from April 2022 to July 2023, representing a total duration of 15 months. Laboratory data were extracted from the hospital’s electronic laboratory information system, and all eligible blood culture isolates identified during the study period were included in the analysis. To avoid duplication, only the first bacterial isolate recovered from each patient was included in the analysis. Repeat blood cultures yielding the same organism from the same patient were excluded. Contaminants identified based on standard microbiological criteria, taking into account the identity of the organism, the number of positive blood culture sets, and the clinical and laboratory interpretation as per the microbiology laboratory, were excluded.

Goroka Provincial Hospital (6°05’S 145°23’E) is a regional referral hospital located in Goroka, Eastern Highlands, Papua New Guinea. The hospital provides healthcare services to an estimated population of approximately 600,000 people, the majority of whom resdide in rural communities with limited access to safe water and adequate sanitation (11). The facility is one of the five national AMR surveillance sites in Papua New Guinea supported through the UK aid – funded Fleming Fund project and contributes routinely collected microbiology data to the national AMR surveillance program.

### 2.2 Laboratory procedures

#### 2.2.1 Bacterial isolation and testing

Blood culture specimens received by the laboratory from clinical wards were incubated at 37 °C in the BACTEC FX 40 Blood Culture System (Becton Dickinson, New Jersey, USA) and processed after flagging for microbial growth. Specimens that did not flag after the 5-day set equipment protocol were reported as negative. Positive specimens were subcultured on blood agar, MacConkey agar, and chocolate blood agar and incubated at 37 °C for 18–24 h. Based on colonial morphology and Gram stain, bacterial identification was done using Phoenix M50 NID and PID panels (Becton Dickinson, New Jersey, USA), while antimicrobial susceptibility testing (AST) to various antibiotic agents was done using quality-controlled disc diffusion technique according to the European Committee on Antimicrobial Susceptibility Testing (EUCAST) guidelines (12). Discrete distinct bacterial colonies isolated from the culture media were tested against various antibiotic agents that are commonly used. The standard panel of antibiotics tested included first-line agents, penicillin (1 μg), cefoxitin (30 μg), chloramphenicol (30 μg), erythromycin (15 μg), trimethoprim-sulfamethoxazole (1.25/23.75ug), tetracycline (30 μg), ampicillin (10 μg), pefloxacin (5 μg), azithromycin (15 μg), amoxicillin-clavulanate (30 μg), gentamicin (10 μg), and ciprofloxacin (5 μg). The cefoxitin (30 μg) disc was used to screen for methicillin resistant *Staphylococcus aureus* (MRSA) with isolates showing a zone of inhibition of <22 mm reported as MRSA (13).

#### 2.2.2 Detection of extended Spectrum β-lactamase

Isolates reported as ceftriaxone resistant during routine testing (inhibition zones of <22 mm) were screened for Extended Spectrum β-lactamase (ESBL) production using the double-disc synergy test (DDST). A disc of ceftriaxone (30 μg), a third-generation cephalosporin antibiotic, was placed 20 mm apart from an amoxicillin-clavulanic acid (30 μg) disc on a lawn culture of the ceftriaxone resistant isolate on Mueller-Hinton agar (Becton Dickinson, New Jersey, USA). ESBL production was inferred when the zone of inhibition was enhanced on the clavulanic acid-containing disc zone presenting with a characteristic “keyhole” shaped zone (14, 15). Additional antibiotics were tested based on a cascade AST testing and reporting strategy implemented by Goroka Provincial Hospital microbiology laboratory. These antibiotics included meropenem (10 μg), ceftazidime (10 μg), piperacillin-tazobactam (30/6 μg), amikacin (30 μg), tobramycin (10 μg) and chloramphenicol (30 μg).

The AST results were recorded and reported as per the facility AST recording and reporting standard operating procedures.

#### 2.2.3 Quality control

Blood cultures were collected using standard aseptic procedures as defined in the hospital specimen collection guide. For adults, one blood culture set consisting of BD BACTEC Plus Aerobic/F and Plus Anaerobic/F bottles was collected, with a recommended blood volume of 8–10 mL per bottle. For paediatric patients, BD BACTEC PEDS Plus/F bottles were used, with the blood volume collected according to the manufacturer’s recommended volume based on the patient’s age and weight. Following collection, blood culture bottles were maintained and transported at room temperature from the clinical wards to the microbiology laboratory for processing. Blood volume adequacy was assessed upon receipt in the laboratory and documented as part of routine quality control. Bottles with inadequate blood volume were recorded and managed according to the laboratory’s established specimen rejection criteria.

*Staphylococcus aureus* American Type Culture Collection (ATCC) 29,213 and *E. coli* ATCC 25922 were used as antimicrobial susceptibility testing quality control strains. Quality control for the phenotypic ESBL assay was performed using the keyhole (double-disc synergy) method. The assay was interpreted based on the characteristic synergistic “keyhole” pattern. The laboratory participates in external quality assessment programs, including the Fleming Fund EQAsia EQA and the Pacific Pathology Training Centre (PPTC EQA), consistently achieving an overall performance exceeding 90%.

### 2.3 Statistical analysis

Patient demographics and bacterial identification and antimicrobial susceptibility data were retrieved from Senaite (NARALABS) LIMS and exported to WHONET through BacLink. Analysis was done using WHONET version 5.6 and IBM SPSS Statistics version 26.0 (IBM Corp, NY, USA). Descriptive statistics were used to summarize patient characteristics, bacterial isolates, and antimicrobial resistance patterns. Continuous variables, such as age, were summarized using median and interquartile ranges (IQR), while categorical variables such as sex and antimicrobial susceptibility patterns were presented as frequencies and percentages. Comparisons of categorical variables were performed using the χ^2^ or Fisher’s exact test as appropriate. Statistical significance was set at α = 0.05, and 95% confidence intervals (CI) were reported where appropriate. To avoid clustered observations, only the first bacterial isolate recovered from each patient was included in the analysis. Antimicrobial susceptibility percentages were generated using WHONET based on AST results for each organism-antimicrobial combination.

## 3 Ethical considerations

This study used de-identified laboratory data with no direct patient contact. Ethical approval was obtained from the Eastern Highlands Provincial Health Authority Research and Ethics Committee (01/2025). Additional authorization was granted by the Goroka Provincial Hospital.

## 4 Results

### 4.1 Study population and blood culture yield

A total of 1,276 blood culture samples were collected from patients with clinically suspected BSI during routine clinical care. The overall blood culture contamination rate was approximately 3%. A total of 40 contaminated blood cultures were not further processed. The 141 (11.4%, 95% CI 9.6–13.2) blood cultures that flagged positive for microbial pathogen were analyzed to confirm the bacterial species and determine the antimicrobial susceptibility (Figure 1).

**Figure 1:**
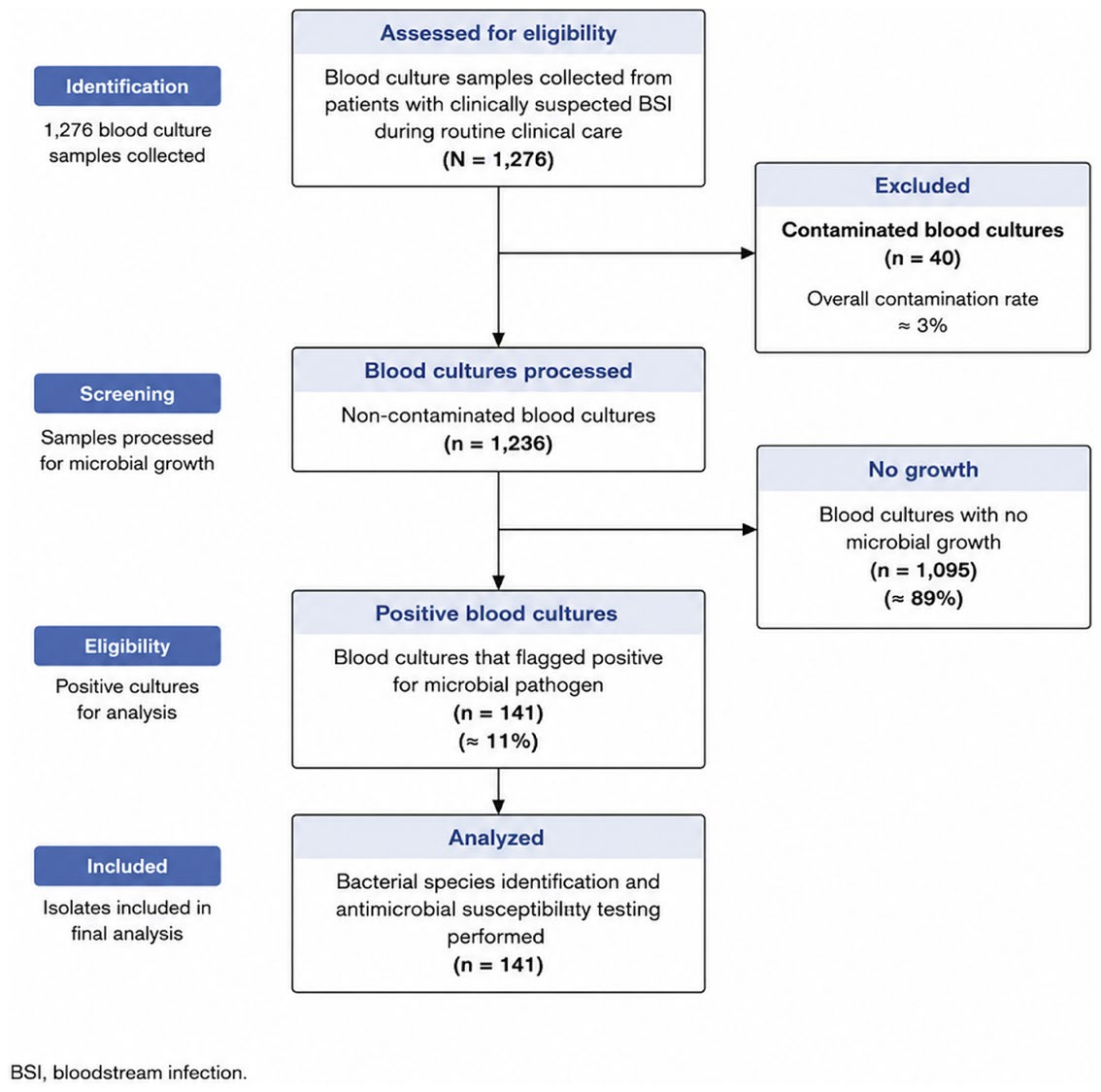
Study population flow diagram for blood culture.

### 4.2 Patient characteristics and antimicrobial exposure

Among the 1,276 paediatric and adult patients, 646 (50.6%, 95% CI 47.8–53.3) were male, and the median age was 19 years (IQR 6–33). More than half of the patients (745/1276, 58.4%, 95% CI 55.7–61.1) had received antibiotics prior to blood culture collection. Of the 745 patients on antibiotcis, ceftriaxone (203, 27.2%, 95% CI 24.0–30.5) was the most commonly used monotherapy, followed by chloramphenicol (88, 11.8%, 95% CI 9.5–14.3), penicillin/amoxicillin (58, 7.8%, 95% CI 6.0–10.0), trimethoprim-sulfamethoxazole (18, 2.4%, 95% CI 1.4–3.8), and ciprofloxacin (14, 1.9%, 95% CI 1.0–3.1). The most frequently administered combination therapies were ceftriaxone - gentamicin (46, 6.2%, 95% CI 4.5–8.2) and chloramphenicol - metronidazole (40, 5.4%, 95% CI 3.9–7.3).

### 4.3 Bacterial distribution

Of the total blood culture specimens processed (*n* = 141) for pathogen isolation and AST, majority were from the emergency department (60, 42.6%, 95% CI 34.3–51.2), followed by pediatric ward (50, 35.5%, 95% CI 27.7–43.9), and medical ward (17, 12.1%, 95% CI 7.2–18.6) with the least from obstetrics and gynecology unit, surgical ward, and special care nursery (each 1, 0.7%, 0.0–3.9). Bacterial isolation was significantly associated with prior antibiotic exposure. Patients who had received antibiotics before blood culture collection were less likely to have a bacterial pathogen isolated than those without prior antibiotic exposure (32/733, 4.4% vs. 109/503, 21,7%, *χ*^2^ = 88.35, df = 1, *p* = 5.39*10^−21^).

Among the bacteria isolated, Gram-negative bacteria (102, 72.3%, 95% CI 64.2–79.5) were isolated nearly three times more frequently than Gram-positive bacteria (39, 27.7%, 95% CI 20.5–35.8). *S.* Typhi (72, 70.6%, 95% CI 60.8–79.2), *E. coli* (9, 8.8%, 95% CI 4.1–16.1) and *K. pneumoniae* (5, 4.9%, 95% CI 1.6–11.1) were the most frequently isolated Gram-negative bacteria. Other Gram-negative bacteria isolated included *Proteus mirabilis*, *Proteus vulgaris*, *A. baumannii*, *Leclarcia* species, *Providencia* species, *Pseudomonas aeruginosa* and *Shigella* species.

*Staphylococcus aureus* (23, 59.0%, 95% CI 42.1–74.4) and *S. pneumoniae* (11, 28.2%, 95% CI 15.0–44.9) were the predominant Gram-positive bacteria isolated, while *Streptococcus pyogenes*, *Streptococcus dysgalactiae*, *Streptococcus anginosus*, and *Streptococcus agalactiae* had a low isolation frequency (Table 1).

**Table 1:** Distribution of bacterial species isolated from blood cultures (*N* = 141).

Bacterial isolate	*n* (%, 95% CI)
*Salmonella* Typhi	72 (51.1, 42.5–59.6)
*Staphylococcus aureus*	23 (16.3, 10.7–23.3)
*Streptococcus pneumoniae*	11 (7.8, 4.0–13.5)
*Escherichia coli*	9 (6.4, 3.0–11.7)
*Klebsiella pneumoniae*	5 (3.6, 1.2–8.0)
*Salmonella* species	3 (2.1, 0.4–6.1)
*Proteus mirabilis*	2 (1.4, 0.2–5.0)
*Shigella* species	2 (1.4, 0.2–5.0)
*Enterobacter cloacae complex*	2 (1.4, 0.2–5.0)
*Streptococcus anginosus*	2 (1.4, 0.2–5.0)
*Burkholderia cepacia* complex	1 (0.7, 0.02–3.9)
*Acinetobacter baumanii* complex	1 (0.7, 0.02–3.9)
*Proteus vulgaris*	1 (0.7, 0.02–3.9)
*Leclarcia* species	1 (0.7, 0.02–3.9)
*Providencia* species	1 (0.7, 0.02–3.9)
*Pseudomonas* species	1 (0.7, 0.02–3.9)
*Streptococcus dysgalactiae*	1 (0.7, 0.02–3.9)
*Shigella flexneri*	1 (0.7, 0.02–3.9)
*Streptococcus pyogenes*	1 (0.7, 0.02–3.9)
*Streptococcus agalactiae*	1 (0.7, 0.02–3.9)

Associations between patient characteristics and the four major bloodstream pathogens (*S.* Typhi, *S. aureus*, *S. pneumoniae,* and *E. coli*) were evaluated among 115 isolates. The remaining 26 of the 141 isolates were excluded from the pathogen-specific comparison. A significant association was observed between patient sex and the bacterial pathogen isolated (*p* = 0.042). *S.* Typhi was isolated more frequently from female patients (42/72, 58.3%, 95% CI 46.1–69.8) than from male patients (30/72, 41.7% 95% CI 30.2–53.9), whereas *S. aureus*, *S. pneumoniae*, and *E. coli* were more frequently isolated from male patients. A significant association was also observed between hospital ward and the bacterial pathogen isolated (*p* = 0.008), with *S.* Typhi accounting for a greater proportion of isolates from the paediatric ward (38/72, 52.8%, 95% CI 40.7–64.7) and emergency department (29/72, 40.3% 95% CI 28.9–52.5). No statistically significant association was observed between patient age group and the bacterial pathogen isolated (*p* = 0.380) (Table 2).

**Table 2:** Distribution of the four major bloodstream pathogens according to patient demographic and clinical characteristics (*N* = 115).

Characteristic	N*	*Salmonella* Typhi (*n* = 72)	*Staphylococcus aureus* (*n* = 23)	*S. pneumoniae* (*n* = 11)	*Escherichia coli* (*n* = 9)	*p*
Age group (years)						
<1	4	1	2	0	1	0.380*
1–4	21	11	4	5	1	
5–14	42	32	5	3	2	
15–44	39	23	10	2	4	
45–64	8	4	2	1	1	
≥65	1	1	0	0	0	
Sex						
Male	59	30	14	7	8	0.042**
Female	56	42	9	3	2	
Hospital ward						
Emergency	40	29	7	2	2	0.008***
Pediatric	52	38	6	5	3	
Medical	15	3	5	3	4	
Surgical	3	1	2	0	0	
Ob and Gyn	1	0	1	0	0	
SCN	4	1	2	1	0	

SCN, special care nursery; Ob and Gyn Obstetrics and Gynaecology; p* and p***Fisher’s xact test; p** χ^2^ test. N* = Only the four major pathogens were included in this comparison; the remaining 26 of the 141 bacterial isolates were excluded from the pathogen-specific analysis.

### 4.4 Antimicrobial resistance patterns

A total of 141 isolates were tested against the selected antimicrobial agents, including *S.* Typhi (*n* = 72), *S. aureus* (*n* = 23), *S. pneumoniae* (*n* = 11), *E. coli* (9) and other bacterial species (*n* = 26).

Among these, *S.* Typhi was the only bacteria that met the standard requirement for antibiogram reporting threshold (*n* = 30 isolates). However, we reported AST results of all bacteria isolated due to their clinical significance. The results indicated that the majority of the isolates were susceptible to the standard panel of antibiotics tested (50–100%). Specifically, *S.* Typhi showed susceptibility to trimethoprim-sulfamethoxazole, ceftriaxone, ampicillin, ciprofloxacin, meropenem, and azithromycin (97–100%). However, nearly half of *S.* Typhi isolates tested against chloramphenicol were resistant (45%).

*Staphylococcus aureus* isolates comprising MRSA and Methicillin Susceptible *Staphylococcus aureus* (MSSA), were susceptible to erythromycin, tetracycline, trimethoprim-sulfamethoxazole, and chloramphenicol (87–100%). Of the 23 *S. aureus* isolates, 14 (61%) were identified as MRSA and demonstrated resistance to flucloxacillin, ampicillin, and penicillin. The remaining MSSA isolates were largerly susceptible to these antibiotics (90–100%). Among the *S. pneumoniae*, approximately 50% of the isolates were resistant to trimethoprim-sulfamethoxazole and tetracycline but mostly susceptible to chloramphenicol (64%) and penicillin (73%).

Among the few isolates tested, *E. coli* showed susceptibility to gentamicin (100%) but resistance to ceftriaxone (33%), trimethoprim-sulfamethoxazole (67%), amoxicillin-clavulanic acid (67%) and ciprofloxacin (78%). Other bacterial isolates tested for antimicrobial susceptibility, including *K. pneumoniae,* were resistant to amoxicillin-clavulanic acid (100%), ceftriaxone (50%), trimethoprim-sulfamethoxazole (54%), gentamicin (10%), and ciprofloxacin (65%). *E. coli* and *K. pneumoniae* isolates that showed reduced susceptibility to ceftriaxone were further tested for ESBL (Table 3).

**Table 3:** Antimicrobial susceptibility profiles of major bloodstream pathogens isolated from blood cultures at Goroka Provincial Hospital, Papua New Guinea.

Bacteria	Antibiotic	Resistant, n/N (%)	95% CI
Gram-negative			
*Salmonella* Typhi (*n* = 72)	Ceftriaxone	0/72 (0.0)	0.0–5.0
	Ampicillin	0/72 (0.0)	0.0–5.0
	Ciprofloxacin	2/72 (2.8)	0.3–9.7
	Meropenem	0/72 (0.0)	0.0–5.0
	Azithromycin	2/72 (2.8)	0.3–9.7
	SXT	0/72 (0.0)	0.0–5.0
	Chloramphenicol	33/72 (45.8)	32.7–58.0
*Escherichia coli* (*n* = 9)	Gentamicin	0/9 (0.0)	0.0–33.6
	Ceftriaxone	3/9 (33.3)	7.5–70.1
	SXT	6/9 (66.7)	29.9–92.5
	AMC	6/9 (66.7)	29.9–92.5
	Ciprofloxacin	7/9 (77.8)	40.0–97.2
Gram-positive			
*Staphylococcus aureus* (*n* = 23)	Erythromycin	3/23 (13.0)	2.8–33.6
	Tetracycline	3/23 (13.0)	2.8–33.6
	SXT	3/23 (13.0)	2.8–33.6
	Chloramphenicol	14/23 (60.9)	38.5–80.3
	Flucloxacilin	14/23 (60.9)	38.5–80.3
	Ampicilin	14/23 (60.9)	38.5–80.3
	Penicilin	14/23 (60.9)	38.5–80.3
*Streptococcus pneumoniae* (*n* = 11)	Tetracycline	6/11 (54.5)	23.4–83.3
	Chloramphenicol	4/11 (36.4)	10.9–69.2
	Penicilin	3/11 (27.3)	6.0–61.0
	SXT	6/11 (54.5)	23.4–83.3

*N* represents the number of isolates tested for each bacterial species. Resistance is presented as resistant isolates/isolates tested (n/N), percentage, and 95% confidence interval. Phenoptypically ESBL-producing isolates were identified according to EUCAST interpretive criteria. SXT, trimethoprim-sulfamethoxazole; AMC, amoxicillin-clavulanic acid.

### 4.5 Phenotypic ESBL detection

Among the few isolates tested, ESBL production was detected in three of the six *E. coli* isolates and four of the six *K. pneumoniae* isolates (Figure 2). Phenotypically ESBL-positive *E. coli* showed resistant to ceftazidime (80%) and chloramphenicol (60%) but susceptible to meropenem (100%), piperacillin-tazobactam (100%), and amikacin (100%). In contrast, phenotypically ESBL-positive *K. pneumoniae* isolates demonstrated susceptible to tobramycin (95%), ceftazidime (100%), piperacillin-tazobactam (100%), amikacin (100%), meropenem (100%), but resistant to chloramphenicol (50%).

**Figure 2:**
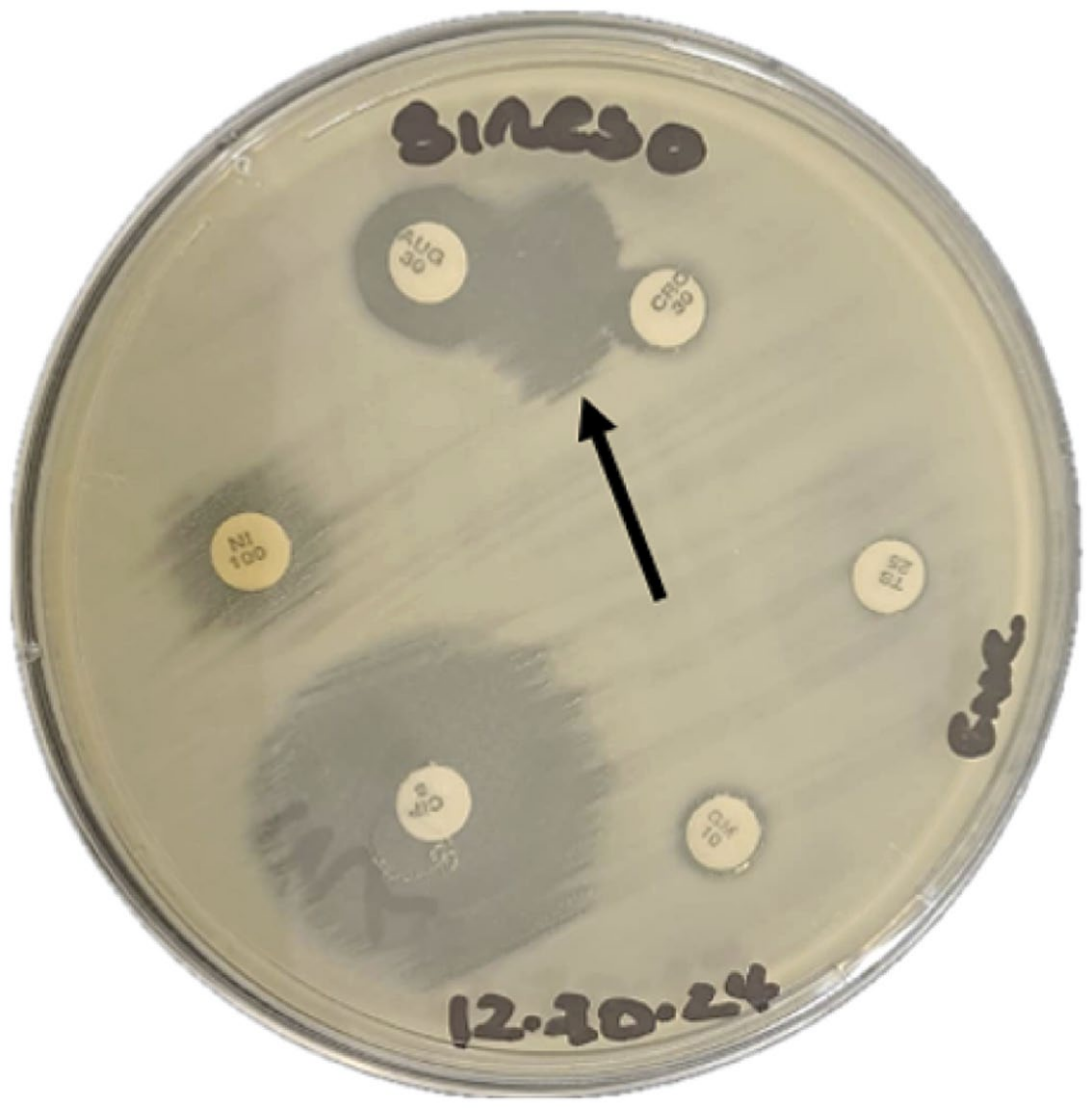
Double disc synergy test (DDST): Keyhole-shaped zone of inhibition (arrow pointed) between the ceftriaxone (CRO,30 μg) and amoxicillin-clavulanic acid (AUG 30 μg) discs placed 20 mm apart, indicating ESBL production by the test isolate; 12–20-24 testing date.

### 4.6 Associations between antibiotic exposure and resistance phenotypes

The associations between prior antimicrobial exposure and selected resistance phenotypes are presented in Tables 4, 5. Among the 12 *E. coli* and *K. pneumoniae* isolates evaluated for phenotypic ESBL production, no significant association was observed between prior ceftriaxone exposure and ESBL production (*p* = 0.558) (Table 4). Similarly, prior chloramphenicol exposure was not significantly associated with chloramphenicol-resistant *S.* Typhi isolates (*p* = 0.402) (Table 5).

**Table 4:** Association between prior ceftriaxone exposure and ESBL detection.

Prior ceftriaxone exposure	*N* = 12	ESBL		*p*-value*
		Positive (*n* = 7)	Negative (*n* = 5)	
Yes	5	2	3	0.558
No	7	5	2	

*Fisher’s exact test.

**Table 5:** Association between chloramphenicol exposure and chloramphenicol-resistant *Salmonella* Typhi.

Prior chloramphenicol exposure	*N* = 72	Chloramphenicol		*p*-value*
		Resistant (*n* = 32)	Susceptible (*n* = 40)	
Yes	24	9	15	0.402
No	48	23	25	

*Pearson chi-square test.

## 5 Discussion

Globally, sepsis is one of the leading causes of death across all age groups and a major focus for disease prevention, antimicrobial stewardship, and early intervention programs (1, 2). In the present study, we aimed to determine the prevalence, bacterial spectrum, antimicrobial susceptibility patterns, and distribution of bloodstream pathogens among patients attending Goroka Provincial Hospital. *S.* Typhi was the predominant bacterial cause of bloodstream infection, followed by *S. aureus*, *S. pneumoniae*, and *E. coli*. Overall, the majority of the isolates were susceptible to most of the antibiotics tested, however, more than half of the *S.* Typhi isolates were resistant to chloramphenicol, while among the isolates tested, a few *E coli* and *K. pneumoniae* strains were phenoptypically ESBL positive.

To the best of our knowledge, this is among the first studies in Papua New Guinea presenting surveillance-based analysis of patient-level BSI data, integrating routine laboratory diagnostics with antimicrobial resistance profiles to inform clinical and public health decision-making.

The blood culture positivity rate (11.1%) observed in the present study was lower than the 15–30% range commonly reported in healthcare facilities across LMICs (2, 5, 10). The low positivity rate could be attributed to patients’ prior exposure to antibiotic therapy before visiting the healthcare facility, reducing the bacterial recovery load by suppressing bacterial growth before sample collection (2, 5). Although the blood culture contamination rate was within the internationally accepted quality benchmarks, contaminated cultures may have also contributed to a reduction in the number of specimens available for pathogen identification (2, 10). Other contributing factors may include differences in specimen collection practices, blood volume inoculated into culture bottles, timing of specimen collection and transportation, and variation in patients’ case mix (5, 10).

The high isolation frequency of Gram-negative bacteria in blood culture specimens in this study is consistent with findings from other similar studies conducted in Brazil, Bangladesh, Indonesia, Thailand, Vietnam, and Ethiopia, that reported Gram-negative bacteria as the most common bacterial pathogen isolated in BSI cases (4, 9, 15–19). The predominance of *S.* Typhi observed in our study may indicate typhoid fever is the main cause of community-acquired BSI in this setting. Our findings mirror observations from other Pacific Island countries such as Fiji and Samoa, where surveillance and hospital-based studies have shown that *S.* Typhi represented the largest proportion of Gram-negative bacteria isolated from positive blood cultures (20). Although Gram-negative bacteria remain the key etiological agent associated with sepsis and BSI, the genus and species may vary. Similar studies conducted in other regions have shown that *K. pneumoniae* and *E. coli,* were the most common Gram-negative bacteria isolated in BSI (5, 16, 17).

The high frequency of *S.* Typhi isolation observed in the present study may be linked to several factors. A significant proportion of the patient cohort resides in rural areas where access to safe drinking water, improved sanitation, and hygiene (WASH) services remains limited in many communities. Although WASH-related factors were not directly assessed in the present study, these conditions are well-recognized risk factors for typhoid transmission and could plausibly contribute to the continued transmission of *S.* Typhi in PNG (11, 19). In addition, genomic and epidemiological surveillance studies from the Pacific region have demonstrated the persistence of locally circulating *S.* Typhi lineages with periodic increases in case numbers, that could be supporting ongoing endemic transmission rather than sporadic outbreaks alone (20, 21). Second, the healthcare-based nature of case ascertainment may have influenced the distribution of organisms recovered in this study, as blood cultures are more likely to be obtained from patients presenting with prolonged fever or clinical features suggestive of systemic infection. In this setting, clinicians preferentially collect and submit blood cultures to the laboratory based on the presence of gastrointestinal manifestations or prolonged fever and epidemiological risk over other clinical presentations of infection, as these are hallmark indicators of systemic *S.* Typhi dissemination. Consequently, this pattern of clinical sampling may have introudcued selection bias and could partly explain the frequency of *S.* Typhi recovered in our study; however, this potential effect could not be quantified (16, 22). Finally, high cases of typhoidal *Salmonella* in our study was seen in pediatric patients, an observation that aligns with what has been reported in other LMICs (18, 20). Exposure to contaminated environments during outdoor activities and poor nutritional status resulting in impairment of the immune system may potentially influence susceptibility to typhoid infection in this patient group. Moreover, in many Pacific Island countries and territories (PICTs), including PNG, limited inclusion of typhoid conjugate vaccine in routine immunization programmes may represent a potential factor contributing to susceptibility among children. Furthermore, microbial detection remains biased as children with febrile illness continue to be a target population in healthcare settings compared to adults where differences in healthcare-seeking behavior and timing of presentation could potentially influence the likelihood of blood-culture confirmation,in adults compared with children (19, 20, 22, 23). The high proportion of MRSA among *S. aureus* isolates recovered in our study indicates that MRSA was frequently detected among the bloodstream isolates examined. Although the study did not distinguish between community- and hospital-acquired infections, several factors may contribute to this finding. These include prolonged circulation of MRSA within healthcare facilities, suboptimal infection prevention and control (IPC) practices, high empirical antimicrobial use, and limited routine microbiological surveillance to guide targeted therapy. Similar challenges have been reported in resource-limited settings, where constrained laboratory capacity and inconsistent antimicrobial stewardship programmes have been proposed as potential contributors to the persistence and transmission of resistant *S. aureus* strains in resource-limited settings (1, 24). The frequent detection of MRSA among the isolates examined underscores the potential value of strengthening routine laboratory surveillance, IPC measures, and antimicrobial stewardship programmes in this setting.

In addition to the high typhoidal *Salmonella* burden, *S. aureus* and *S. pneumoniae* were the second and third most frequently isolated bacterial pathogens, respectively. Our findings are consistent with reports from elsewhere, including PICTs which have shown that *S. aureus* is among the leading Gram-positive bacteria isolated in community and hospital-onset BSI (25). Other PICTs including Fiji and Samoa have also found low isolation rate of *S. pneumoniae* (26). Similar findings have been reported in other LMICs such as Brazil, Bangladesh, Indonesia, Vietnam, and Ethiopia (4, 9, 15–17). Earlier literature especially from Africa, Brazil, and PICTs show high *S. pneumoniae* carriage and infection rates among adults and children (27–30). With the introduction and scale-up of pneumococcal conjugate vaccine (PCV) programs in most of these countries, there has been a subsequent reduction of invasive pneumococcal disease and carriage, especially of vaccine serotypes among young children (26, 27). Additionally, low isolation rates of *S. pneumoniae* observed in the present study and across comparable literature could be attributed to pre-hospital antibiotic exposure and differences in clinical sampling protocols. Notably, the divergent isolation rates between inpatients and outpatients may partly reflect differences in blood-culture sampling practices, including potential undesrsampling of patients presenting with pneumonia, particularly those managed in outpatient settings (27, 29). Regardless, the impact of PCV has changed the epidemiology of invasive pneumococcal disease, especially among young children, with herd protection reducing infections among the adult population (2, 29).

Isolation of Gram-negative Enterobacterales, specifically *E. coli* and *K. pneumoniae* in the present study was low contrary to what has been reported globally. In LMICs, non-typhoidal Gram-negative bacteria have been reported in hospital-based surveillance and studies focusing on hospital-acquired infections as the most common bacteria associated with BSI (16, 25). The isolation variation observed in our study compared to those from LMICs may partly be explained by the prior empirical use of antibiotics either as self-medication or as routine practice in lower-tier health facilities in the region among patients subsequently referred to Goroka Provincial Hospital for specialized care. The observed lower recovery of bacterial pathogens among patients with prior antibiotic exposure may also be influenced by differences in illness severity, age, ward of admission, referral status, duration of illness and the timing of antibiotic administration relative to blood culture collection. The dominance of Gram-negative Enterobacterales and the observed resistant patterns in the present study may contribute to the myriad of AMR cases as Pacific region countries document worrying rise and transmission of resistant microorganisms (21, 25).

The antimicrobial susceptibility profile of *S.* Typhi observed in the present study has important implications for the empirical management of BSI in Papua New Guinea. Although only *S.* Typhi met the recommended minimum isolate threshold for antibiogram reporting, susceptibility testing of all bloodstream isolates provided valuable local evidence to guide clinical practice. Most *S.* Typhi isolates were susceptible to trimethoprim-sulfamethoxazole, ampicillin, ceftriaxone, ciprofloxacin, azithromycin, and meropenem, indicating that these agents remain effective treatment options in this setting. These findings were similar to what was has been reported in India, Bangladesh, Malawi, and Kenya (31). However, nearly half of the isolates were resistant to chloramphenicol, contrasting with reports of restored chloramphenicol susceptibility in several Asian countries following reduced clinical use of the drug (24). This regional discrepancy can be attributed to variations in antimicrobial stewardship and selection pressure. While majority of the Asian countries largely abandoned chloramphenicol in favor of fluoroquinolones and cephalosporins removing the evolutionary pressure, chloramphenicol has remained the primary drug of choice in the present study setting. The continuous clinical and empirical use of this drug has likely maintained a constant selective pressure, favoring the preservation of plasmid-mediated resistance genes within the local *S.* Typhi pool (32, 33). These findings emphasize that antimicrobial resistance patterns are context-specific and highlight the importance of sustained local laboratory surveillance. Additionally, locally generated antimicrobial susceptibility data are essential for informing empirical treatment guidelines, strengthening antimicrobial stewardship programs, and monitoring emerging resistance trends in PNG, rather than relying solely on regional or global resistance patterns.

The high proportion of MRSA observed in our setting is comparable to findings from hospital-based studies conducted in Vietnam, India, Tanzania, and Uganda, where the MRSA isolation rate was more than 50% (24). As expected, the MRSA demonstrated resistance to penicillin, ampicillin, and flucloxacillin, preserving susceptibility to erythromycin, trimethoprim-sulfamethoxazole, tetracycline, and chloramphenicol, similar to observations from several African and PICTs (24, 31). Findings from routine laboratory surveillance support the empirical use of non-beta-lactam anti-staphylococcal agents for suspected MRSA infections in both hospital and community-acquired. Additionally, these findings reinforce the need to strengthen infection prevention and control (IPC) strategies and antimicrobial stewardship (AMS) programs to reduce MRSA transmission and limit the emergence and spread of AMR. Contrary to *S. aureus* susceptibility, half of the *S. pneumoniae* isolates tested were resistant to commonly used agents such as tetracycline and trimethoprim-sulfamethoxazole. This aligns with global trends reported across the Pacific, Asia, and Africa, that could be linked to widespread community use, especially amongst the paediatric population (4, 31). Susceptibility to penicillin and chloramphenicol reported in our setting against *S. pneumoniae* may suggest that beta-lactams are still effective for pneumococcal infections. However, the susceptibility rates in our setting (73 and 64%, respectively) and the declining rates globally are a major concern, especially in cases of invasive pneumococcal disease.

*Escherichia coli* and *K. pneumoniae* isolates were resistant to trimethoprim-sulfamethoxazole, amoxicillin-clavulanic acid, ciprofloxacin, and ceftriaxone, which are among the first-line drugs used in our setting for hospital and community-acquired bacterial infections. Similar observations have been reported in other countries, especially LMICs, showing global expansion of ESBL-producing Enterobacterales (4, 14, 16). In the present study, phenotypically ESBL-positive *E. coli* and *K. pneumoniae* accounted for approximately half of the isolates tested, underscoring the ongoing challenge posed by ESBL-producing microorganisms. Despite the observed ESBL phenotype, these isolates remained susceptible in-vitro to piperacillin-tazobactam, meropenem, and amikacin. These agents may represent important therapeutic options, particularly for severe infections; however, their use may need to consider antimicrobial stewardship, treatment cost, sustainability, and the potential selection and emergence of antimicrobial resistance (31).

### 5.1 Strengths and limitations of the study

The major strength of this study is the use of laboratory-confirmed bloodstream isolates with AST from both adults and pediatric patients across a wide range of clinical units. This broad representation enhances the applicability of the findings to routine clinical care, AMR surveillance, and antimicrobial stewardship efforts. Nevertherless, our study had several limitations. Laboratory surveillance was conducted continuously throughout the study period, reflecting routine clinical practice without interruptions in data collection. Although the study spanned more than 12 months, seasonal variation in bloodstream infections and AMR patterns was not assessed. Future studies should evaluate potential seasonal trends to determine whether temporal variations influence the distribution of bloodstream pathogens and their antimicrobial resistance profiles in PNG. The facility-based design of this study may limit generalizability of the findings to other healthcare settings, including community and regional facilities, where the distribution of circulating pathogens, AMR patterns, and prescribing practices may differ. Furthermore, because the study relied on routinely collected laborartory surveillance data, it was not possible to distinguish community-acquired from hospital-acquired bloodstream infections, limiting interpretation of the epidemiology and transmission dynamics of the pathogens identified. In addition, the present study did not include patient-level clinical outcomes such as in-hospital mortality, treatment failure, duration of hospitalization. Consequently, these outcomes could not be evaluated highlighting the need for future prospective studies to better define the clinical and public health impact of BSI and AMR in PNG. On prior antibiotic exposure, the laboratory-based dataset did not contain sufficient information to determine the specific antibiotic source, duration of therpay, or exact interval between antibiotoic administration and blood collection. A further limitation is that ESBL-producing isolates were identified using routine phenotypic antimicrobial susceptibility testing without a formal ESBL-positive quality control reference strain (ATCC 25218) and molecular confirmation. Therefore, specific AMR genes (CTX-M, SHV, TEM, and OXA) and underlying resistance mechanisms could not be determined highlighting the need to strengthen national genomic surveillance capacity through future investment and collaboration to support AMR surveillance and outbreak investigations. In addition, the small sample size for less frequently isolated pathogens restricted the precision of resistance estimate. However, findings from this study provide baseline data to guide local empirical treatment guidelines, strengthen AMR monitoring and antimicrobial stewardship, using methodology that could be applied in other resource-limited settings.

## 6 Conclusion

*Salmonella* Typhi was the predominant bacterial isolate recovered from blood cultures in our study, particularly among infants and children younger than 14 years. These isolates remained susceptible *in vitro* to most of the commonly used antibiotics, although substantial resistance to chloramphenicol was observed. A high proportion of *S. aureus* isolates were methicillin resistant, while most *E. coli* and *K. pneumoniae* tested were ESBL-producing phenotype. These findings support continued laboratory-based AMR surveillance and locally informed IPC and AMS, including the routine use of facility-specific antibiograms. Integrating antimicrobial use (AMU) and consumption (AMC) data into surveillance systems, together with sustained routine AST, will provide high-quality evidence to inform empirical treatment guidelines, support national AMR action plans, and preserve the effectiveness of existing antibiotic therapies.

## Data Availability

The original contributions presented in the study are included in the article/supplementary material, further inquiries can be directed to the corresponding author/s.
